# Response to correspondence on “B chromosomes of multiple species have intense evolutionary dynamics and accumulated genes related to important biological processes”

**DOI:** 10.1186/s12864-023-09881-6

**Published:** 2023-12-18

**Authors:** Syed F. Ahmad, Guilherme T. Valente, Cesar Martins

**Affiliations:** 1 Department of Structural and Functional Biology, Institute of Bioscience at Botucatu, Sao Paulo State University (UNESP), Botucatu, 18618-689 SP Brazil; 2 Animal Genomics and Bioresource Research Unit (AGB Research Unit), Faculty of Science, Kasetsart University, 50 Ngamwongwan, Chatuchak, Bangkok, 10900 Thailand

The correspondence article by [1] (Ruiz-Ruano and Camacho, 2023) raises a number of important and valid concerns in our previous article [2](Ahmad et al. 2020). We are grateful to commentators for their exquisitely detailed and rigorous comments on our paper. Here, we respond to make corrections recognizing our unintentional mistakes in the paper and address the major comments in the following points.

## 1. Information of sequenced data in our study

We acknowledge the comment by Ruiz-Ruano and Camacho [1] that “The reads deposited in the Sequence Read Archive (SRA) had already been trimmed but it was not specified in the paper”. Here, we would like to inform the readers of our paper [2] that the sequenced data was submitted to SRA as trimmed reads and did not have raw data available. We apologize for the inconvenience caused by the unavailability of the raw data due to HPC technical maintenance. However, the mentioned fact is not so dramatic since an initial data analysis concerning the quality of reads by FastQC toolkit and data filtering would show that the mentioned library was already filtered. We believe that the preprocessing filtering step of raw data has no significant effect on utilizing this data resource for further research. Therefore, this sample should be treated as trimmed data and appropriate filtering/trimming options should be considered by retrievers as per the requirements of downstream analysis.

## 2. Inaccuracies in our supplementary figures and tables of [2]

In response to the errors mentioned in “misleading datasets” by Ruiz-Ruano and Camacho [1], we acknowledge these errors, and therefore we provide the following details:

We rechecked the missing accession numbers in the coverage plots of Figures S7 and S8 [2] and enlist these IDs with relevant information (Table 1).

**Table 1:** Missing data from Figures S7 and S8 [2]

Transcript ID of reference transcriptome	0B reads	1 (2) B reads	1(2)B/0B	Annotation	Gene/Repeat Type	Log_2_ ratio
***Astyanax mexicanus***						
ARA0AAA39YG16EM1.b.am.1	727	14,308	19.68	novel_gene	Gene	4.3
ARA0AAA100YB14EM1.b.am.1	10	160	16	Wu	Gene	4
ARA0AAA109YO19EM1.b.am.1	9	73	8.11	(CAA)n	DNA_repeats	3.02
ARA0AAA114YD18EM2.b.am.1	7	128	18.29	Dldh	Gene	4.19
ARA0ABA18YP03EM1.b.am.1	9	63	7	unknown	unknown	2.81
ARA0ABA86YJ16EM1.b.am.1	3	39	13	(TTGT)n	DNA_repeats	3.7
ARA0ABA110YF05EM1.b.am.1	11	111	10.09	unknown	unknown	3.34
ARA0AGA13YG24EM1.b.am.1	89	472	5.3	unknown	unknown	2.41
***A. correntinus***						
ARA0AAA7YM13EM1.b.am.1	1	152	152	(AATA)n	DNA_repeats	7.25
ARA0AAA8YO20EM1.b.am.1	102	143	1.4	ERV1A-CPo_I-int	DNA_repeats	0.49
ARA0AAA105YI15EM1.b.am.1	277	1496	5.4	Tars	Gene	2.43
ARA0ABA109YD04EM1.b.am.1	311	400	1.29	(AAT)n	DNA_repeats	0.36
ARA0AAA27YN14EM1.b.am.1	572	3585	6.27	unknown	unknown	2.65
ARA0ABA89YC04EM1.b.am.1	118	933	7.91	unknown	unknown	2.98
ARA0AAA69YE12EM1.b.am.1	1005	1612	1.6	I-3_DR	DNA_repeats	0.68
ARA0ABA32YK17EM1.b.am.1	70	376	5.37	unknown	unknown	2.43

We further clarify that the transcriptome assembly of *A. mexicanus* (used as reference for both *A. mexicanus* and *A. correntinus*) was retrieved from http://genotoul-contigbrowser.toulouse.inra.fr:9099/ngspipelines/data/1651096bdb/analysis/96cf6c918c/contigs.fa.gz. This transcriptome assembly was submitted by Hinaux et al. [3] in the web browser http://genotoul-contigbrowser.toulouse.inra.fr:9099. The transcriptome assembly of *L. migratoria* (used as reference for *A. flavolineata*) was retrieved from NCBI database (accession ID: GDIO00000000.1).

In addition, we clarify that “The information regarding *A. mexicanus* and *A. correntinus* has been mistakenly interchanged”. The *A. mexicanus* mappings in Table 3 of Ahmad et al. [2] correspond to *A. correntinus* and vice versa. In addition, the column of “2B” actually correspond to “1B” reads in the Supplementary Dataset 3 [2]. We are sorry that such mistakes could have caused confusion for readers, but at the same time we reinforce that the BMC Genomics makes available contacts of the authors that should be reached in any doubt while accessing data.

## 3. Low reproducibility and data normalization

In their comment, the commentators pointed out a core issue of low reproducibility in the results of *A. correntinus*, mainly in the transcriptome-based coverage ratio analysis. We appreciate the commentators’ efforts in their attempts to reproducing the results and detecting many false positive genes. They emphasize data normalization due to higher sequencing coverage in B+(1B) dataset, which we neglected in our article [2], and they offer a rigorous explanation of the approaches employed for avoiding false-positive genes on B chromosomes.

Meanwhile, we would like to point out that due to the scarcity of a gold-standard protocol to study B chromosomes, uncertain mapping parameters, and lack of rigorous statistical significance criteria for normalization, it is difficult to rule out the possibility of false-positive genes/sequences on B chromosomes and data validation. Therefore, specialized bioinformatic tools with detailed step-by-step protocols with given instructions on adapting the appropriate technique are essential for valid detection of genes on B chromosomes. This can further benefit the B chromosome research community and also allow beginner bioinformaticians or biologists with basic computational skills to avoid the technical errors. Nevertheless, due to the variety of experimental systems, providing a universal strategy for each approach is not feasible. Therefore, it is recommended to identify the most optimal parameters for one’s specific study in order to get the best results.

## 4. Additional issues and corrections

Here, first we would like to sincerely apologize for the unintended errors in our published work:

1. We acknowledge that some of the references were mistakenly not cited in the given context.
2. We apologize for mistakenly not citing the “2019 bioRxiv preprint” [4] reference in the statement “evolutionary success of the B chromosome lies on its gene contents”.
3. We acknowledge that schematic model shown as Fig. 9 in our manuscript was partially adopted from idea presented in Martis et al. [5]. Although we have cited Martis et al. [5] in our work [2], we apologize for the unintentional omission of this mention in figure caption.
4. We agree with the concern of utilizing data from microdissected B chromosome sequences may have generated bias as discussed in the comment. We want to clarify that although we applied stringent filtering criteria for this analysis, we recognize the limitations of low coverage data and the pseudo-scaffolding strategy must be considered before drawing any strong conclusion from this data. However, this was not a mistake since that was the data which was generated and retrieved from NCBI at that time.
5. Apart from admitting the errors mentioned, we respectfully disagree with some of the conceptual critiques on our paper’s speculations, as stated in the discussion section of commentary.

Concerning the definition of B chromosome, we do not attempt to define “B chromosome”, but only to contrast the characteristics of A (regular chromosomes) and B by noting that in most of the cases reported, Bs are unpaired unlike A. Although an initial definition for universal properties of B chromosomes was proposed in 1993 (1st B Chromosome Conference, Spain (Beukeboom 1994 [6])) we see that Bs exhibit diverse behavior across different organisms and have generated extensive debates among scientists, as registered during the 5th B Chromosome Conference (Serbia, 14–17 October, 2023 - https://5thbcc.com/).

In regards to the comment of null hypothesis, we acknowledge that the phenomenon of selfish transmission has not been observed in any of the three species we studied, but our aim was to study whether B chromosomes tend to accumulate certain sequences that could confer them a transmission advantage. It is important to clarify that our paper does not include a “null hypothesis”; instead, we establish a research question in the introduction to delineate the objectives of our study, which had an exploratory goal.

We do not agree with comment that our claim of “migration of substantial genomic regions from A to B chromosomes via transpositions, duplications and rearrangements” lacks data support. Our paper presents genome-based detection, FISH mapping, qPCR validation and literature review as supporting evidence for our statement.

We disagree with the commentary point of tagging one of our paper’s sentences as “an anti-Darwinian post-adaptive statement”. In our paper, we suggest that B chromosomes may have acquired a tendency to gain sequences for their own maintenance in the cell. We want to clarify that, this mention in our study does not contradict Darwinism in any way; and we further encourage future studies to test whether these sequences on B are accumulated by chance randomly or by selection. To avoid the discrepancy regarding our original paper’s sentence we revised the sentence as follow:

It seems that the movement of sequences onto the B chromosome after its formation per se is likely not selected for, but instead these events may be undirected, and certain sequences, once moved, could be advantageous to the B chromosome.

## Conclusions

The advancement of B chromosome science depends on the continuous development of research methods that contribute to improve existing knowledge and overcome the limitations of previous studies to generate new insights. One example is our study published in 2020, which contributes to the field of B chromosome biology by generating high coverage sequencing datasets and providing insights into detection of sequences located on B chromosome of different species. Nevertheless, our study (Ahmad et al. 2020) [2] was not without errors and certain technical limitations in its methodological approach, as pointed out by commentators [1] (Ruiz-Ruano and Camacho, 2023). We acknowledge the potential bias, and appreciate this feedback of several corrections. We do believe that there are still many challenges and gaps in analytical approaches to fully understand the B chromosome genomic content that need to be addressed by future studies. It is recommended to establish well-defined and gold-standard protocols, especially bioinformatic tools, that can ensure better reproducibility of results and reliability of the methodology employed for studying the complexity of B chromosomes.

## Data Availability

Not applicable.
